# Building staff capability, opportunity, and motivation to provide smoking cessation to people with cancer in Australian cancer treatment centres: development of an implementation intervention framework for the Care to Quit cluster randomised controlled trial

**DOI:** 10.1007/s10742-022-00288-6

**Published:** 2022-09-28

**Authors:** Annika Ryan, Alison Luk Young, Jordan Tait, Kristen McCarter, Melissa McEnallay, Fiona Day, James McLennan, Catherine Segan, Gillian Blanchard, Laura Healey, Sandra Avery, Sarah White, Shalini Vinod, Linda Bradford, Christine L. Paul

**Affiliations:** 1 School of Medicine and Public Health, College of Health, Medicine and Wellbeing, University of Newcastle, Callaghan, NSW Australia; 2 Hunter Medical Research Institute, John Hunter Hospital, Level 4 West, New Lambton Heights, Newcastle, NSW Australia; 3 Priority Research Centre for Cancer Research, Innovation and Translation, University of Newcastle, 2308, Callaghan, NSW Australia; 4 Hunter Cancer Research Alliance, John Hunter Hospital, New Lambton Heights, Newcastle, NSW Australia; 5 Calvary Mater Newcastle, Corner Edith and Platt Streets, Waratah, NSW 2289 Australia; 6 St Vincent’s Hospital Sydney, 390 Victoria Street, Darlinghurst, NSW 2010 Australia; 7 Cancer Council Victoria, Melbourne, VIC Australia; 8 School of Population and Global Health, Centre for Health Policy, The University of Melbourne, MelbourneMelbourne, VIC Australia; 9 South Western Sydney Local Health District, Elizabeth Street, Liverpool, NSW 2170 Australia; 10 Department of Health Quitline, 615 St Kilda Rd, Melbourne, VIC 3004 Australia; 11 Cancer Therapy Centre, Liverpool Hospital, Liverpool, NSW Australia; 12 South Western Sydney Clinical School and Ingham Institute for Applied Medical Research, Liverpool, NSW Australia; 13 The Alfred, 55 Commercial Rd, Melbourne, VIC 3004 Australia; 14 School of Nursing and Midwifery, University of Newcastle, Callaghan, NSW Australia

**Keywords:** Implementation, Intervention mapping, Behavioural framework, Cancer care, 3As model of care, Smoking cessation

## Abstract

Few rigorous studies provide a clear description of the methodological approach of developing an evidence-based implementation intervention, prior to implementation at scale. This study describes the development, mapping, rating, and review of the implementation strategies for the Care to Quit smoking cessation trial, prior to application in nine cancer services across Australia. Key stakeholders were engaged in the process from conception through to rating, reviewing and refinement of strategies and principles. An initial scoping review identified 21 barriers to provision of evidence-based smoking cessation care to patients with cancer, which were mapped to the Theoretical Domains Framework and Behaviour Change Wheel (BCW) to identify relevant intervention functions. The mapping identified 26 relevant behaviour change techniques, summarised into 11 implementation strategies. The implementation strategies were rated and reviewed against the BCW Affordability, Practicality, Effectiveness and cost-effectiveness, Acceptability, Side-effects/safety, and Equity criteria by key stakeholders during two interactive workshops to facilitate a focus on feasible interventions likely to resonate with clinical staff. The implementation strategies and associated intervention tools were then collated by form and function to provide a practical guide for implementing the intervention. This study illustrates the rigorous use of theories and frameworks to arrive at a practical intervention guide, with potential to inform future replication and scalability of evidence-based implementation across a range of health service settings.

**Supplementary Information:**

The online version contains supplementary material available at 10.1007/s10742-022-00288-6.

## Introduction

Cigarette smoking among people diagnosed with cancer is associated with a host of adverse treatment-related outcomes such as increased toxicity and complications (Clark et al. 2007; Cowen et al. 2010; Merkow et al. 2009; Sørensen et al. 2002; van der Voet et al. 1998), cancer recurrence (Kenfield et al. 2011; Lammers et al. 2011; van Imhoff et al. 2016; Wyszynski et al. 2014) and cancer-specific and overall survival (Kelly et al. 2011; Smith et al. 2019; Zingg et al. 2011). Compared to quitting at diagnosis, continued smoking doubles the risk of death and can half median survival time for lung cancer patients (Cataldo et al. 2010). Numerous studies have demonstrated that smoking cessation after a cancer diagnosis can improve treatment outcomes, including survival (Al-Mamgani et al. 2014, Amato et al. 2015, Barnett et al. 2020, Chen et al. 2019, Karam‐Hage et al. 2014, Nia et al. 2005, Roach et al. 2016, Tabuchi et al. 2017, Warren et al. 2020). Other than tumour site and stage at diagnosis, abstinence from smoking is the strongest predictor of survival in patients with cancer who have ever smoked (Karam‐Hage et al. 2014).

The National Comprehensive Cancer Network (NCCN) (Shields et al. 2015), emphasised the importance of smoking cessation for cancer patients and establishment of an evidence-based standard of care including pharmacotherapy (e.g., Nicotine Replacement Therapy; NRT) and behavioural therapy (recommendation of 4 + sessions of 10–30 min over 12 weeks from a professional trained in smoking cessation in addition to brief physician advice). Australia’s peak multidisciplinary oncology society (i.e., COSA) for health care professionals (HCPs) recommends a three-step brief 3As (ask, advice, act) model of care implementable within oncology services that includes clear referral pathways for smoking cessation pharmacotherapy and multi-session behavioural therapy (COSA 2020). Embedding smoking cessation care requires a multidisciplinary approach and there is a call in the literature (Young et al. in press) for implementation trials to improve the standardisation and up-scale of evidence-based smoking cessation care in oncology services.

Prior to implementation of a multi-component trial, the UK Medical Research Council stipulates that the early development of strategies through reviewing literature and pilot work can increase the likelihood of successful implementation (Craig et al. 2008). A range of smoking cessation interventions are evident (e.g., electronic medical record (eMR) embedding, NRT opt-out approach), yet findings from within Australia indicate that staff-level barriers are most difficult to change, particularly when relating to addressing attitudinal and motivational barriers. Results from a national survey of 295 Australian oncologists show that the majority of respondents indicated a strong preference for smoking cessation care to be managed by other HCPs, with only 4% of medical oncologists and 0% of radiation oncologists preferring to manage care themselves (Day et al. 2018). A 2-page smoking cessation pathway increased screening and referral of up to 92% of cancer patients (Young et al. in press), yet variability across sites was influenced by HCPs perceived level of responsibility to intervene. Standardisation of care requires staff commitment, awareness, role allocation, and system-level changes for seamless integration (Geerligs et al. 2018). HCPs have also reported low self-efficacy to deliver brief smoking advice and patient resistance as impediments toe delivery of smoking cessation care (Day et al. 2018).

This methodological study describes the steps and approaches used to develop the implementation strategies for the Care to Quit cluster randomised controlled trial (Paul et al. 2021), which was established in collaboration with key stakeholders who directed the development from conception through mapping, rating, review and refinement of strategies and principles. This involved a structured plan to comprehensively address barriers to provision of smoking cessation care using the Theoretical Domains Framework (TDF) (French et al. 2012), a determinant framework, and the Behaviour Change Wheel (BCW) (Michie et al. 2011), which is a behavioural model aimed at characterising interventions within a system, matching the behaviour with intervention functions and policy categories (Michie et al. 2011). The TDF is used to describe determinants of behaviours, categoried into fourteen domains, to identify a target behaviour, and guide behaviour change interventions from planning through to evaluation (e.g. *what* to change). The BCW is a behavioural framework used to design behaviour change impementation strategies (e.g. *how* to change). The core element of the framework is based around the capability, opportunity, and motivation to enact the target behaviour (COM-B), influenced by nine possible intervention functions and seven policy types. It comprises three key steps for designing behavioural interventions: understanding the target behaviour, identify intervention options, and identify content, and implementation options. The application of the BCW APEASE criteria to the resultant implementation strategies produces a focus on actions which are expected to be feasible and to resonate with health professionals. Further, the standardisation of complex health service interventions by its functions (principles or objectives) is superior to standardisation by its form (mode of delivery) (Perez Jolles et al. 2019). This paradigm shift encourages prospective consideration of how implementation interventions can be adapted to improve intervention-context-fit, prior to being implemented at scale. For this paper, implementation interventions is defined as “interventions designed to change clinical practice behaviour and improve the uptake of evidence into practice” (French et al. 2012).

## Methods

### Aims and objectives

The aim of this study is to describe the scientific and methodological approach used to develop the implementation strategies of the Care to Quit trial, targeting staff at Australian cancer treatment services. The main objective is two-fold: first to reach consensus amongst key stakeholders about the barriers in their health service, and then to elicit perceptions of implementable strategies to overcome such barriers.

### Design and setting

The Care to Quit stepped wedge cluster randomised trial is funded by the National Health and Medical Research Council and administered by The University of Newcastle, Australia. The trial is managed by a committee comprising academic researchers, governing authorities, and health service providers with behavioural, cancer and smoking cessation expertise (Paul et al. 2020, 2021) and will be rolled out across nine Australian cancer treatment services in metropolitan and regional New South Wales (NSW) and Victoria (VIC).

### Materials and processes

The research team at the University of Newcastle (herein referred to as the “team”) conducted a scoping review to identify barriers and facilitators to the provision of smoking cessation care within cancer centres. The review was informed by prior work conducted by the team through staff surveys (Day et al. 2018), including patient interviews (Sherwood et al. 2017) using Australian and international smoking cessation guidelines (COSA 2020; Royal Australian College of General Practitioners 2021). From this, the team identified a list of barriers, which were summarised into themes. The development of the implementation intervention included several evaluation stages. We used the APEASE criteria to rate and review the implementation strategies with a group of key stakeholders prior to finalising the implementation strategies. The methods and results of the development process have been structured according to the BCW recommended stages and steps for implementation interventions (Michie et al. 2011) – see Table 1*,* and in accordance with the Template for Intervention Description and Replication (TIDIER) checklist (Additional File 1).

**Table 1:** Development of the Care to Quit implementation intervention

Step 1	Identification of barriers to provision of smoking cessation care in cancer centres via a scoping review, informed by previous work by the team, with barriers summarised into themes
Step 2	Mapping of barriers to the components of the Theoretical Domains Framework and COM-B, and the intervention functions of the BCW Taxonomy 1
Step 3	Double coding of the mapping, with discrepancies solved by discussion using a consensus-based approach
Step 4	Rating of intervention functions against the APEASE criteria, resulting in selection of BCW intervention functions considered effective for each COM-B component
Step 5	Review of possible behaviour change techniques (BCT’s) suitable to the selected intervention functions. This was done by application of APEASE criteria to select BCT’s to elicit behaviour change among cancer care providers, with discrepancies solved by consensus, summarised into implementation strategies
Step 6	Rating and review of implementation strategies with key stakeholders via two video-meetings using APEASE criteria, with discussions of lower rated strategies resulting in confirmation of strategies following minor adjustments to their descriptions
Step 7	Identification of intervention principles underpinning the implementation intervention, which were then mapped to form and function, and standardised by function
Step 8	Division of implementation strategies into three stages, with the initial stage focusing on building staff capability and motivation, a second stage encompassing identification of site champions, followed by a third stage focusing on identification and implementation of cessation models of care / pathways, supported by a suite of tools and resources, tailored to site context and preferences

## Results

### Stage 1: Understand the behaviour

#### Define the problem in behavioural terms

The behavioural problem was identified as a lack of provision of smoking cessation care among cancer care providers within Australian cancer centres. The findings from the literature indicate that cancer care providers generally report high rates of asking patients about their smoking status, however the provision of advice or assistance for cancer patients to quit smoking is limited (Day et al. 2018; Derksen et al. 2020; Warren et al. 2013a, b; Warren et al. 2013a, b). Few cancer care providers make referrals to cessation support and commonly discuss smoking cessation medications (Day et al. 2018).

#### Select and specify the target behaviour

The target behaviour was identified as delivery of smoking cessation care using the brief Ask-Advise-Act (3As) smoking cessation model of care. The utilisation of the shorter 3As model is consistent with NSW, VIC, and National Comprehensive Cancer Network recommendations for evidence-based cessation support for patients with cancer (Shields et al. 2015), and allows delivery by a wide range of health professionals working in a variety of settings (Kaiser et al. 2018; Vidrine et al. 2013). Using a whole-of-service approach was determined as most suitable as patients often see a range of cancer care providers during their treatment. Service leaders were also considered important key stakeholders to endorse the approach.

The target behaviour requires that all treatment providers involved with the care of the cancer patient (oncologists, nurses, radiation therapists, pharmacists, and allied health professionals) at every consultation should:

- Provide brief smoking cessation care to all cancer patients using the 3As: Ask, Advice, Act smoking cessation model of care. This will involve assessing and recording smoking status; give a personalised description of the specific benefits of smoking abstinence during and after treatment; and endorse use of evidence-based support such as telephone counselling and pharmacotherapy
- Offer referral to Quitline or local cessation support services, which are free of charge
- Prescribe or provide advice on pharmacotherapy options licensed for use in Australia such as Nicotine Replacement Therapy (NRT) or Varenicline (Champix), which are safe during treatment and subsidised via the Pharmaceutical Benefit Scheme (PBS), and
- Monitor progress.

#### Identify what needs to change

The scoping review identified 21 barriers to the provision of smoking cessation care at cancer centres, which were mapped to five of the six COM-B sources of behaviours (*psychological capability, reflective motivation, automatic motivation, physical opportunity, and social opportunity)* and 12 of the 14 TDF components (see Table 2). Barriers were then grouped into five themes identifying what needs to change.

**Table 2:** Intervention mapping of staff barriers to COM-B and TDF components, intervention functions, and BCW techniques

Message theme	Staff barriers to providing smoking cessation care	COM-B components	TDF components	Intervention functions	Behaviour change techniques (BCT taxonomy)
Highlighting the importance of dynamic smoking cessation support for people recently diagnosed with cancer	1) Limited knowledge about the benefits of smoking cessation for treatment outcomes and quality of life for patients with cancer (Charlesworth et al. 2019; Conlon et al. 2017; Cubbin 2016; Sarna et al. 2001; Simmons et al. 2009; Taniguchi et al. 2011; Weaver et al. 2012; Weiss et al. 2020; Wells et al. 2017)	Psychological capability	Knowledge	Education	Information about health consequences Information about emotional consequences Salience of consequences Credible source Comparative imagining of future outcomes Information about other’s approval
	2) Lower preference for providing smoking cessation care during cancer treatment; most oncology providers prefer to provide cessation assistance at the initial consult or following treatment (Day et al. 2018)	Reflective motivation Psychological capability	Intentions Beliefs about consequences Professional role and identity Knowledge Cognitive and interpersonal skills	Education Persuasion Training	Information about health consequences Information about emotional consequences Credible source Comparative imagining of future outcomes Demonstration of the behaviour Instruction on how to perform a behaviour Behavioural practice/rehearsal Framing/reframing Prompts/cues Action planning Habit formation Feedback on behaviour Information about other’s approval Pros and cons
	3) Lower motivation to provide smoking cessation care for current/recent smokers with non-smoking-related cancers (Day et al. 2018; Taniguchi et al. 2011)	Reflective motivation	Intentions Beliefs about consequences Professional role and identity Goals	Education Persuasion	Information about health consequences Comparative imagining of future outcomes Credible source Framing/reframing Generalisation of a target behaviour Prompts/cues Action planning Habit formation Behavioural practice/rehearsal Feedback on behaviour
Incorporating pharmacotherapies in helping cancer patients quit smoking	4) Limited knowledge about the potential interactions between smoking cessation pharmacotherapies and cancer treatments (Day et al. 2018; Luxton et al. 2019)	Psychological capability	Knowledge Memory, attention and decision processes	Education	Information about health consequences Credible source
	5) Lack of availability or accessibility of NRT in some Australian hospital pharmacies (Luxton et al. 2019)	Physical opportunity	Environmental context and resources	Environmental restructuring	Restructuring the physical environment Restructuring the social environment Feedback on behaviour
	6) Lack of knowledge about the availability, cost and insurance coverage of pharmacotherapy treatment options (Sarna and Bialous 2016a, b; Sarna and Bialous 2016a, b)	Psychological capability	Knowledge Memory, attention and decision processes	Education Training	Information about social and environmental consequences Credible source Prompts/cues
	7) Perception that smoking cessation counselling would be ineffective (Conlon et al. 2017; Sarna et al. 2001; Schnoll et al. 2006; Sutton et al. 2013)	Reflective motivation Psychological capability	Beliefs about consequences Optimism	Education Persuasion	Framing/reframing Credible source Information about health consequences Verbal persuasion about capability Comparative imagining of future outcomes Incompatible beliefs Identity associated with changed behaviour
Increasing general knowledge and awareness of smoking cessation strategies and resources among oncology providers	8) Lack of knowledge and/or training in providing smoking cessation care (Chang et al. 2017; Charlesworth et al. 2019; Conlon et al. 2017; Cubbin 2016; Day et al. 2018; Derksen et al. 2020; Goldstein et al. 2012; Luxton et al. 2019; Movsisyan et al. 2012; Price et al. 2019; Sarna et al. 2001; Tong et al. 2020; Warren et al. 2013a, b; Warren et al. 2013a, b; Warren et al. 2015; Weaver et al. 2012; Weiss et al. 2020)	Psychological Capability	Knowledge Cognitive and interpersonal skills	Training Education	Instruction on how to perform the behaviour Demonstration of the behaviour Behavioural practice/rehearsal Prompts/cues Feedback on behaviour
	9) Limited experience or skills in providing smoking cessation support (Chang et al. 2017; Conlon et al. 2017; Cubbin 2016; Gosselin et al. 2011; Lina et al. 2016; Luxton et al. 2019; Sarna et al. 2001; Warren et al. 2013a, b; Weiss et al. 2020)	Psychological capability	Cognitive and interpersonal skills	Education Training	Instruction on how to perform the behaviour Demonstration of the behaviour Behavioural practice/rehearsal Prompts/cues Feedback on behaviour
	10) Lack of available resources/referral pathways (Chang et al. 2017; Charlesworth et al. 2019; Coovadia et al. 2020; Day et al. 2018; Luxton et al. 2019; Price et al. 2019; Singer et al. 2019; Tong et al. 2020; Warren et al. 2013a, b; Warren et al. 2013a, b; Warren et al. 2015; Weiss et al. 2020)	Physical opportunity	Environmental context and resources	Environmental restructuring Enablement	Restructuring the physical environment Problem solving Prompts/cues Feedback on behaviour
	11) Limited knowledge of smoking cessation resources, referral pathways, or methods for prescribing cessation pharmacotherapy (Coovadia et al. 2020; Lina et al. 2016; Luxton et al. 2019; Ma et al. 2016; Price et al. 2019; Singer et al. 2019; Weaver et al. 2012; Wells et al. 2017)	Psychological capability	Knowledge Memory, attention and decision processes	Education	Problem solving Prompts/cues Instruction on how to perform the behaviour Demonstration of the behaviour Feedback on behaviour
Addressing perceived challenges in discussing smoking cessation with cancer patients	**12)** Belief that patients lack the motivation to quit (Charlesworth et al. 2019; Conlon et al. 2017; Gosselin et al. 2011; Ma et al. 2016; Price et al. 2019; Sarna et al. 2001; Simmons et al. 2009; Warren et al. 2013a, b; Weaver et al. 2012; Weiss et al. 2020)	Reflective motivation	Beliefs about capabilities Optimism	Education Persuasion	Credible source Framing/reframing Feedback on behaviour Information about other’s approval Prompts/cues
	13) Overreliance on overt indicators of smoking status that would overlook many smokers, particularly recent quitters, e.g. tobacco scent (Charlesworth et al. 2019; Weiss et al. 2020)	Automatic motivation Psychological capability	Reinforcement Knowledge	Persuasion Education	Credible source Framing/reframing Prompts/cues Generalisation of target behaviour Habit formation Behavioural practice/rehearsal Feedback on behaviour
	14) Belief that patients would be resistant to smoking cessation treatment (Chang et al. 2017; Day et al. 2018; Derksen et al. 2020; Luxton et al. 2019; Ma et al. 2016; Price et al. 2019; Schnoll et al. 2006; Simmons et al. 2009; Warren et al. 2013a, b; Warren et al. 2013a, b; Warren et al. 2015; Weaver et al. 2012)	Reflective motivation Psychological capability	Beliefs about capabilities Optimism Cognitive and interpersonal skills	Education Persuasion Training	Credible source Framing/reframing Demonstration of the behaviour Instruction on how to perform the behaviour Behavioural practice/rehearsal Information about other’s approval Feedback on behaviour
	15) Fear that smoking cessation intervention could increase feelings of stress, guilt and blame or be viewed as judgemental by cancer patients (Chang et al. 2017; Charlesworth et al. 2019; Cubbin 2016; Lina et al. 2016; Luxton et al. 2019; Movsisyan et al. 2012; Sarna et al. 2001; Simmons et al. 2009; Weiss et al. 2020; Wells et al. 2017)	Reflective motivation Psychological capability	Beliefs about consequences Cognitive and interpersonal skills	Education Persuasion Training	Credible source Framing/reframing Demonstration of the behaviour Instruction on how to perform the behaviour Behavioural practice/rehearsal Information about other’s approval Feedback on behaviour
	16) Belief that patients are too overwhelmed or overloaded by competing demands of cancer diagnosis or treatment to discuss smoking cessation (Charlesworth et al. 2019; Ma et al. 2016; Price et al. 2019; Wells et al. 2017)	Reflective motivation Psychological capability	Beliefs about consequences Cognitive and interpersonal skills	Education Persuasion Training	Credible source Framing/reframing Demonstration of the behaviour Instruction on how to perform the behaviour Behavioural practice/rehearsal Information about other’s approval Feedback on behaviour
	17) Perception that discussing smoking cessation could interfere with rapport or therapeutic relationship (Charlesworth et al. 2019; Price et al. 2019)	Reflective motivation Psychological capability	Beliefs about consequences Cognitive and interpersonal skills	Education Persuasion Training	Credible source Framing/ reframing Demonstration of the behaviour Instruction on how to perform the behaviour Behavioural practice/rehearsal Information about other’s approval Feedback on behaviour
	18) Low confidence or perceived inability for assisting patients to quit smoking (Charlesworth et al. 2019; Conlon et al. 2017; Day et al. 2018; Derksen et al. 2020; Price et al. 2019; Sarna et al. 2001; Warren et al. 2013a, b; Warren et al. 2013a, b; Weaver et al. 2012)	Reflective motivation Psychological capability	Beliefs about consequences Beliefs about capabilities Optimism Cognitive and interpersonal skills	Education Persuasion Training	Credible source Verbal persuasion about capability Framing/reframing Demonstration of the behaviour Instruction on how to perform the behaviour Behavioural practice/rehearsal Feedback on behaviour Focus on past success
Addressing institutional challenges to providing smoking cessation care in oncology centres	19) Lack of institutional engagement or management support (Coovadia et al. 2020; Luxton et al. 2019; Tong et al. 2020; Wells et al. 2017)	Social opportunity	Social influences	Environmental restructuring	Restructuring the social environment Social comparison Information about other’s approval Feedback on behaviour Credible source
	20) Perceived lack of time for addressing smoking cessation (Chang et al. 2017; Charlesworth et al. 2019; Conlon et al. 2017; Coovadia et al. 2020; Day et al. 2018; Derksen et al. 2020; Lina et al. 2016; Luxton et al. 2019; Ma et al. 2016; Price et al. 2019; Schnoll et al. 2006; Singer et al. 2019; Sutton et al. 2013; Warren et al. 2013a, b; Warren et al. 2013a, b; Warren et al. 2015; Weiss et al. 2020)	Reflective motivation Psychological capability	Beliefs about capabilities Professional role and identity Intentions Cognitive and interpersonal skills	Education Persuasion Training	Information about health consequences Comparative imaging of future outcomes Framing/reframing Demonstration of the behaviour Instruction on how to perform the behaviour Behavioural practice/rehearsal Feedback on behaviour
	21) Lack of role clarity or perceived responsibility for smoking cessation care; belief that duty falls to other health care providers (Charlesworth et al. 2019; Conlon et al. 2017; Luxton et al. 2019; Movsisyan et al. 2012; Price et al. 2019; Sutton et al. 2013; Wells et al. 2017)	Reflective motivation	Professional role and identity Intentions	Education Persuasion	Framing/reframing Persuasion about capability Identification of self as role model Social comparison Information about other’s approval Feedback on behaviour

### Stage 2: Identify intervention and policy options

When rated against the six APEASE criteria (*acceptability, practiability, effectiveness, affordability, side-effects, and equity*), the intervention functions *education, training, persuasion, environmental restructuring, and enablement* were considered appropriate for the implementation intervention, while the intervention functions of *incentivisation, restrictions, coercion* and *modelling* were excluded. The selection was informed by the BCW guide which describes which intervention functions are considered effective for each of the COM-B components (see column 3 in Table 2). There were no additional policy categories identified as relevant for this study, and fiscal measures and legislation were not within the scope of the trial.

### Stage 3. Identify content and implementation options

The team considered all possible techniques and arrived at 26 unique BCT’s considered promising to elicit behaviour change among cancer care providers. These are listed in the BCT column in Table 2*.* Relevant techniques derived from the BCW Taxonomy 1 (Michie et al. 2013), with a focus on techniques that were considered as implementable in busy clinical settings involving a variety or professional groups *(goals and planning; feedback and monitoring; shaping knowledge; natural consequences; comparison of behaviour; associations; repetition and substitusion; comparison of outcomes; antecedents; identity; and self-belief*). The five groups that were not feasible and appopriate within the scope of the trial, and thus not included, were: social support; reward and threat; regulation; scheduled consequences; and covert learning.

### Identify mode of delivery

The team made a consensus-based decision to divide the 12-month intervention phase of the implementation intervention into three stages to allow for the foundations of each site to be in place, prior to moving into the more training and resource intensive part of the implementation intervention. The three-phased implementation intervention will focus on increasing staff capability, opportunity, and motivation to provide smoking cessation care to cancer patients at every consultation, involving all treatment providers.

To improve capability, we will educate clinicians about the consequences of continued smoking among patients with cancer and train them in the 3As cessation model of care. *Educating* clinicians will involve BCT’s such as *presenting information about the health consequences from a credible source* and *highlighting the salience* of these consequences. *Training* clinicians in smoking cessation care will involve *instruction on how to perform the behaviour*, *demonstrations of the target behaviour*, and *behavioural practice/rehearsal* with *role-play* exercises and *prompts and cues* to facilitate the target behaviour in patient encounters.

To improve motivation, we will use BCTs including *information about others’ approval*, *reframing* smoking behaviour with patients and verbal *persuasions* about capability for helping patients quit. This will include *positive testimonials from patients and clinicians* designed to vicariously expose cancer care providers who may not have experienced success in cessation care to the possible benefits and the value in persisting. Presenting evidence about the benefits of smoking cessation will *enable* to motivate clinicians.

To improve opportunity, BCTs including *restructuring the physical and social environment* will be employed to improve referral and prescription pathways. Increasing buy-in from service leadership may also help to enhance opportunities for smoking cessation care among clinicians.

The implementation intervention will involve multiple components and modes of delivery, noting that some strategies will overlap and may be impacted or adjusted due to COVID-19 requirements or implications, for examples face-to-face components may be delivered via webinars or other online modalities, and delivery will be, as mentioned earlier, tailored to local contexts (see Table 2). Having a flexible delivery approach is anticipated to enhance staff participation despite challenges resulting from the COVID-19 pandemic. It should also be noted that Telehealth alternatives have recently become more routine in oncology practice and the provision of smoking cessation care may be provided using this modality (Burbury et al. 2021; Paterson et al. 2020).

*In stage 1*, outreach visits will be delivered by a behavioural scientist with smoking expertise, a cancer clinician, and a Quitline counsellor, with evidence-based messages tailored to the professional role of the clinician and the patient group (tumour type, treatment outcomes) around benefits of cessation, multi-session behavioural support services offered via services such as Quitline, use of pharmacotherapy during cancer treatment, and importance of timing and framing of cessation messages. Visits will be conducted according to site preferences, with subsequent visits focusing on review of recent consultations and discussions around challenging cases.

*Stage 2* will focus on identifying site champions and formation of a champion team at each site. It is envisaged that these teams comprise staff interested in driving and facilitating the implementation of the 3A smoking cessation model of care in their service.

*In stage 3*, site champions identified in Stage 2 will be supported by the team through a range of mechanisms including regular and ad hoc phone or video calls, with the aim to identify how to implement the 3As smoking cessation model of care within existing patient pathways to increase opportunities for smoking cessation support. Concurrently, a suite of evidence-based implementation strategies tailored to the needs of the individual site will be employed to build staff capability (e.g. skills, knowledge and confidence) to actively use cessation models of care / pathways and provide smoking cessation support for patients with cancer. The method of providing pharmacotherapy will vary as not all centres will be able to provide this directly to outpatients, thus the treating clinician may write a prescription that the patient will take to the pharmacy or a referral to the patient’s General Practitioner. Training will be supported by educational brochures for clinicians and patients, and reminders such as desktop prompts and scripts. Utilisation of patient journey maps may also occur, which is a common way to showcase the steps in the patient journey and highlight complexities around inter-departmental processes of care.

### Results of rating and reviewing the Care to Quit implementation intervention against the APEASE criteria prior to implementation at scale

To ensure successful delivery of the implementation intervention, the eleven implementation strategies were rated and reviewed using the APEASE criteria with a group of nine principal and associate investigators prior to implementation. Stakeholders included two oncology service providers in NSW, one representative from the Cancer Institute NSW (CINSW), one academic with experience of smoking cessation and cancer care implementation trials, one tobacco treatment specalist consultant psychologist, one cancer systems innovation manager, two representatives from VIC Quitline, and a nurse practitioner at a cancer treatment centre. Strategies that were rated lower were discussed as a group with barriers and potential solutions noted in a summary sheet. The results of this process determined the *Affordability, Practicality, Effectiveness and cost-effectiveness, Acceptability, Side-effects/safety,* and *Equity* of the implementation intervention approach, prior to implementation at scale. Scheduling of time to perform the implementation strategies by one or more clinicians was generally rated lower than strategies that could be utilised anytime or without specific clinician input such as desktop prompts and scripts, CINSW online training modules and educational videos from known experts/clinicians (see Table 3).

**Table 3:** Results of rating and reviewing the implementation intervention with key stakeholders using APEASE

Strategy	Mean score	Stakeholder feedback
*STAGE 1 – to engage with staff, assess current practices/perceptions and build motivation to provide smoking cessation care*		
Outreach visits short group and individual meetings to present the case for smoking cessation care	33.83/45	Stakeholders raised some concerns around affordability, practicality, acceptability and equity of outreach visits, with potential solutions identified as having a proactive and flexible delivery approach guided by site availability and preferences, scheduling of group and individual visits in liaison with clinical champions conducted using a range of delivery modalities (face to face, videoconference etc.), promotion of outreach visits by clinical staff, and linking in with pre-arranged departmental meetings
Educational videos from known experts/clinicians persuasive videos with multiple content options designed to highlight the importance of smoking cessation care and motivate staff – embedded in outreach visits	38.50/45	Not discussed in detail as all criteria rated positively (4–5) by all attendees
Patient testimonial videos videos presenting patient’s perspective designed to motivate staff to provide smoking cessation care – embedded in outreach visits	37.33/45	The practicality of patient testimonial videos was discussed, with recommendations to keep videos short (2–3 min). Clinical members of the expert group raised concerns around the acceptability and value of patient videos in an often time-poor clinic setting, however discussions also highlighted that such videos can be powerful to help clinicians feel more comfortable to raise patient smoking status and to respond to patient expectations if the clinician is also a smoker
*STAGE 3 – to build capability (e.g. knowledge, skills, confidence) to provide cessation care*		
Educational videos from known experts/clinicians this broadly covers a range of online learning programs and methods	38.67/45	Not discussed in detail as nearly all criteria were rated positively (4–5) by attendees
Training delivered to site champions following the ‘train-the-trainer’ model, we’ll be training site champions on how to train fellow staff to provide smoking cessation care	36.33/45	Concerns around practicality and cost-effectiveness of site champion training were highlighted from both a researcher and clinician perspective, due to difficulties in coordinating training and challenges to incorporate training into an already busy clinical schedule The workload of site champions was highlighted. The expert group identified that site champions need to be motivated to be involved and that site champions would be best identified through an expression of interest process rather than individuals who are nominated for the role To increase feasibility, the research team would make themselves available to support site champions and provide regular check-ins. It was suggested to use webinar style training as these can be more cost-effective and supplement face-to-face training without losing effectiveness
Training deliverd by site champions to other staff brief face-to-face training that champions will deliver to other staff with our team helping to organise and implement	32.00/45	The team highlighted affordability, equity, practicality, effectiveness, and side effects/safety of training delivered by site champions to other staff, as this would require time outside of their clinical role and require a backfill, with sustainability challenges considering staff turnover and equity challenges for time-poor staff to participate in face to face modalities A better option would be for champions to facilitate self-paced training and inclusion of training in staff orientation packs, with additional support from the research team acting as a buffer to coordinate and get staff engaged with this somewhat complex process There is a risk that site champions may be influenced by previous experiences and attitudes and put their own slant on the training messages which would impair the standardisation of the training. Further, problems can arise when site champions are asked questions that are outside the remit of their training Possible solutions would be to create a standardised training protocol, and readily available content expertise via research team or content resource, avoiding misinformation being gathered by the end-user
Online training modules this broadly covers a range of online learning programs and methods	37.00/45	The team identified effectiveness and acceptability of online training modules as potentially challenging, as some staff do not engage with static online training, and existing smoking cessation modules are more focused on a population level thus potentially less acceptable for oncology staff An interactive webinar style training was identified as a possible solution
Site specific patient journey maps developed with site champions and tailored to the local context, these maps will demonstrate when and how the 3 aspects of smoking cessation care will be delivered	36.17/45	Concerns around affordability was raised if left to sites to coordinate, however might work better if a staff member from the research team is overseeing this process Practicality concerns in the clinical context included potential for staff to be exposed to multiple patient journey maps for a variety of different projects. This concern was highlighted but a solution was not identified
Educational resources (e.g. brochures for clinicians and patients) this includes leaflets and brochures for staff and patients	38.67/45	While the effectiveness of educational resources for clinicians can be effective, the effectiveness of patient resources are questionable
Online CINSW training modules (nine lessons) to evaluate the CINSW training module for smoking cessation care in cancer services, designed to assist staff to develop knowledge and skills to provide brief cessation care. Module comprise 9 lessons ~ 30 min or 2–7 min per individual lesson	38.00/45	Effectiveness and acceptability were highlighted with concerns similar to general online training e.g. lack of staff engagement however acceptability might be boosted by highlighting the short length of the training (around 30 min in total), rather than indicating that the training involves nine modules
Desktop prompts and scripts to prompt clinicians and facilitate discussions with patients about smoking, the reasons for quitting and referrals to support services	39.00/45	The effectiveness of a scripted smoking cessation care process is not likely to work as patients may not engage with discussions if too scripted, however simplified flowchart prompts of the smoking cessation care process could help clinicians to develop their own scripts for discussing smoking with their patients

*Note: Criteria were rated using a 5-point likert scale where 1* = *lowest score and 5* = *highest score. Max score per variable* = *45. Number of participants rating each variable* = *9*

### Mapping the principle of the implementation intervention by form and function

Using methods identified by Hawe et al. (Hawe et al. 2004), the research team identified five core implementation intervention principles, which were to (1) educate clinicians about the benefits of smoking cessation care for treatment outcomes and quality of life, (2) increase provision of smoking cessation care: at all stages of treatment, (3) increase the provision of smoking cessation care for all patients who smoke regardless of smoking related cancer and for recent quitters, (4) increase provision of smoking cessation care: specifically, using the 3As model of care, and (5) to increase the provision of smoking cessation care: specifically, referrals for evidence-based treatment i.e. combined counselling (Quitline or other referral) and pharmacotherapies (i.e. combination NRT, varenicline).

The research team then mapped each principle by form and function (see Table 4), with implementation interventions standardised by function to provide a more flexible and tailored delivery approach. To facilitate easy, timely and sustained clinical access to intervention material, while also allowing flexible training options, the team developed a project website, hosted on an open-source platform, with built-in site-specific tailoring modalities. Existing content, largely material from the CINSW (Cancer Institute NSW 2021) was identified with permissions sought to use it in the study (and website). Where desired content was unavailable from existing sources, the research team identified new content options to be developed, and worked with key stakeholders to develop these. The approach of the general practitioner was included to showcase and highlight smoking cessation work in the primary health sector and improve continuity of care across primary, tertiary and community sectors.

**Table 4:** Mapping the five principles of the Care to Quit intervention to form and function

Principle of intervention	Standardised by function	Identified format options for tailoring to site context
1. To educate clinicians about the benefits of smoking cessation care for treatment outcomes and quality of life	Work with sites to devise ways to distribute information tailored to local context (literacy, language, culture, learning styles)	CINSW online training module 1 – *Why smoking cessation is important for people with cancer* Educational persuasive videos from known experts and clinicians: to highlight the importance of smoking cessation care and motivate staff – embedded in outreach visits Patient testimonial videos: presenting patient’s perspective designed to motivate staff to provide smoking cessation care – embedded in outreach visits Online factsheets: highlighting the role of the clinician in providing smoking cessation care Tailored information sheet: of cessation benefits to tumour type, treatment outcome, professional role Patient handouts: short and longterm outcomes of smoking cessation
2. To increase provision of smoking cessation care: at all stages of treatment	Provide sites with materials and resources to develop a tailored workshop/training	CINSW online training module 2 – *Your role in smoking cessation for people with cancer*: to help staff to develop knowledge and skills in provision of brief cessation care Clinician videos: demonstrating how to deliver smoking cessation as part of every consultation Patient testimonial videos: presenting patient’s perspective designed to motivate staff to provide smoking cessation care – embedded in outreach visits Patient journey maps (site-specific): developed with site champions & tailored to local context, demonstrating when & how the 3 aspects of smoking cessation care will be delivered Audit & feedback: of clinician provision of smoking cessation care (surveys, case study), to boost motivation
3. To increase the provision of smoking cessation care: for all patient who smoke regardless of smoking related cancer (and for recent quitters)	Provide sites with materials and resources to develop a tailored workshop/training	Reminders Clinician videos: demonstrating how to deliver smoking cessation as part of every consultation Patient testimonial videos: presenting patient’s perspective designed to motivate staff to provide smoking cessation care – embedded in outreach visits Audit & feedback: of clinician provision of smoking cessation care (surveys, case study), to boost motivation
4. To increase the provision of smoking cessation care: specifically, using the 3As: ASK, ADVISE, ACT)	Sites to devise ways to distribute information tailored to local context (literacy, language, culture, learning styles)	CINSW online training modules 3–8: *introduce and deliver the 3As brief intervention, barriers & skills practice* Training videos (CINSW & other): showing how oncologists, radiation therapists, nurses, GPs and pharmacists deliver 3As at initial and follow up visits: Clinician videos: demonstrating how to deliver smoking cessation as part of every consultation Role-plays Environmental change: via research team support Smoking status and referral in electronic medical record Project factsheet Example script: for responding to patient resistance Reminders via desktop scripts and prompts: to prompt clinicians and facilitate discussions with patients about smoking, reasons for quitting and referrals to support services
**5.** To increase the provision of smoking cessation care: specifically, referrals for evidence-based treatment i.e. combined counselling (Quitline or other referral) and pharmacotherapies (i.e. combination NRT, varenicline)	Provide sites/champion teams with resources and support to increase provision and develop referral pathways where necessary for prescribing	CINSW online training module 9 – *Smoking cessation supports* Training videos (CINSW & other): around prescribing combined counselling and pharmacotherapy support Patient handouts: information and benefits of combined counselling and pharmacotherapies Reminders via desktop scripts and prompts: to prompt clinicians & facilitate discussions with patients about smoking, reasons for quitting and referrals to support services Example referral pathways: to pharmacy for prescriptions/access, for tailoring to site context Example referral letters: for GPs for prescribing NRT Poster: for patient waiting area, to motivate patients to discuss smoking cessation care with their provider Quitline referral form (online, fax) Smoking cessation badge (for clinician) Factsheets/booklets Smoking status and referral in electronic medical record Audit & feedback: of clinician provision of smoking cessation care (surveys, case study), to boost motivation

## Discussion

This methodological study aimed to describe the theory-driven and evidence-informed process used to develop the Care to Quit implementation intervention in collaboration with key stakeholders. The approach held focus on key barriers impeding provision of smoking cessation care at the cancer treatment services and identifying implementable strategies to overcome such barriers. Collaborative evaluative meetings with key stakeholders ensured that contextual, organisational and staff barriers, particularly motivation and attitudinal barriers, were addressed within three distinct stages of the trial. Mapping the principles of the intervention to the domains of the TDF and relevant COM-B sources of behaviours provided a theoretical basis for characterising targeted behaviours requiring change within the health system context.

Knowledge was identified as the most prominent staff barrier to providing smoking cessation care. Key stakeholders identified that oncology HCPs lack knowledge about the benefits of smoking cessation care, referral pathways, and methods for prescribing pharmacotherapy. The BCW postulates that education, training, and modelling are required to improve knowledge and distinguishes *education* as a form of imparting knowledge while the term *training* is a form of imparting skill (Michie et al. 2011). In the current study, strategies to improve education include expert and patient videos, leaflets, and evidence from studies on the importance of smoking cessation for people with cancer. The mode of delivery was acceptable, but HCPs questioned the sustainability of training due to staff turnover, busy workloads and identifying who would train existing staff in each cancer centre. Indeed, countless tools and platforms have been developed to engage HCPs. The main trial will integrate training through an online platform that will continually be available throughout implementation and in conjoint with local government agencies, has the potential to become an ongoing resource for HCPs throughout Australia.

Motivation and staff attitudes towards smoking cessation care also influenced the acceptability of the implementation strategies. A clear barrier to care resultant from the literature (Chang et al. 2017; Charlesworth et al. 2019; Conlon et al. 2017; Coovadia et al. 2020; Day et al. 2018; Derksen et al. 2020; Lina et al. 2016; Luxton et al. 2019; Ma et al. 2016; Price et al. 2019; Schnoll et al. 2006; Singer et al. 2019; Sutton et al. 2013; Warren et al. 2013a, b; Warren et al. 2013a, b; Warren et al. 2015; Weiss et al. 2020) and evaluation discussions was the perceived lack of time for addressing smoking cessation. Belief that patients lack the motivation to quit (Charlesworth et al. 2019; Conlon et al. 2017; Gosselin et al. 2011; Ma et al. 2016; Price et al. 2019; Sarna et al. 2001; Simmons et al. 2009; Warren et al. 2013a, b; Weaver et al. 2012; Weiss et al. 2020) is commonly reported, while HCPs have often believed that patients would be resistant to smoking cessation treatment (Chang et al. 2017; Day et al. 2018; Derksen et al. 2020; Luxton et al. 2019; Ma et al. 2016; Price et al. 2019; Schnoll et al. 2006; Simmons et al. 2009; Warren et al. 2013a, b; Warren et al. 2013a, b; Warren et al. 2015; Weaver et al. 2012). The trial initially devotes an extended period to engage with HCPs, allowing the researchers to address the motivational and commitment factors that impede care. To overcome the time shortage HCPs face, small group interactive discussions during the initial phase of the Care to Quit trial will provide relevant, tailored, and streamlined education that is anticipated to require a minimal time commitment by being embedded in existing training and engagement opportunities where possible, with key cessation information accessed via optimised administrative systems. Engagement with key leaders and hospital managers early will be vital. The alignment with hospital standards, using peak oncology agencies (CINSW) and services (e.g., Quitline) are strong facilitators for sustainable change (Rankin et al. 2015). Equally important is the engagement with staff from varying roles and levels to ensure that the development and application of strategies is relevant and applicable to each context.

Although many studies have used a theoretical framework and behavioural theory to guide larger implementation trials, few papers provide sufficient guidance about the work conducted to develop the implementation intervention and level of stakeholder engagement throughout the establishment process to allow scalability and transferability across multiple contexts and settings (Campbell et al. 2000). Emerging evidence has identified that standardisation of complex health service interventions by its functions (principles or objectives) is superior to standardisation by its form (mode of delivery), without compromising fidelity, which is a paradigm shift that encourages prospective consideration of how implementation interventions can be adapted to improve intervention-context-fit, prior to being implemented at scale. Sustainable efforts are often those that are valued by clinicians, patients, and the health service overall with knowledge embedded in protocols and pathways that are co-designed by multidisciplinary teams and consumer engagement, aligned with legislative and guideline supported approaches. Building capability and motivation to provide opportunities to practice a new behaviour or skillset takes dedication and time before becoming habit and routine practice. The Care to Quit trial is novel in its early and continuous engagement with key stakeholders in the conceptualisation and development of the implementation strategy, which is envisaged to assist with continuous involvement and value-creation throughout the study.

### Limitations

This study was designed at the time of the COVID-19 worldwide pandemic thus will require flexibility to allow implementation across a range of unforeseen circumstances. Nevertheless, the learnings of this study may guide future developments within and beyond the pandemic landscape and bring light to those that are new to the development of complex behavioural implementation trials.

## Conclusions

This study illustrates the full process from the rigorous use of theories and frameworks to arriving at a practical intervention guide and set of intervention tools for an implementation trial. This work has potential to facilitate knowledge translation and contribute to more rigorously designed health system implementation trials, ultimately relevant to a variety of contextual elements with its flexible tailored implementation approach.

## Supplementary Information

Below is the link to the electronic supplementary material.

Supplementary file1 (DOCX 37 kb)

## Data Availability

The data generated or analysed during this study are included in this published article and its supplementary information files.
