# Supplementary material for: Building staff capability, opportunity, and motivation to provide smoking cessation to people with cancer in Australian cancer treatment centres: development of an implementation intervention framework for the Care to Quit cluster randomised controlled trial
Source: Health Serv Outcomes Res Methodol. 2022 Sep 28:1–33. Online ahead of print. doi: 10.1007/s10742-022-00288-6 (PMC9517978; doi:10.1007/s10742-022-00288-6)
Supplement: Supplementary file 1 — Supplementary file1 (DOCX 37 kb) [file 10742_2022_288_MOESM1_ESM.docx]

**
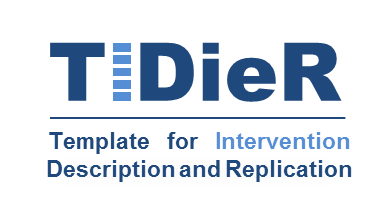
The TIDieR (Template for Intervention Description and Replication) Checklist*:**

Information to include when describing an intervention and the location of the information

| **Item number** | **Item** | **Where located **** | |
| --- | --- | --- | --- |
|  |  | Primary paper  (page or appendix  number) | Other ^†^ (details) |
|  | **BRIEF NAME** |  |  |
| **1.** | Provide the name or a phrase that describes the intervention. | Building staff capability, opportunity, and motivation to provide smoking cessation to people with cancer in Australian cancer treatment centres: development of an implementation intervention framework for the Care to Quit cluster randomised controlled trial. See Title page (page 1). | ______________ |
|  | **WHY** | See Results section of the Abstract (page 3), Introduction section (pages 4-5), Tables 1-4, and the Results section (pages 6-10) |  |
| **2.** | Describe any rationale, theory, or goal of the elements essential to the intervention. |  | _____________ |
|  | **WHAT** |  |  |
| **3.** | Materials: Describe any physical or informational materials used in the intervention, including those provided to participants or used in intervention delivery or in training of intervention providers. Provide information on where the materials can be accessed (e.g. online appendix, URL). | Materials are included in Table 4, referred to in Table 3, and in the text in the Results section under subheading “Mapping the principle of the implementation intervention by form and function”, (page 10). | _____________ |
| **4.** | Procedures: Describe each of the procedures, activities, and/or processes used in the intervention, including any enabling or support activities. | Procedures are included in Table 3, Table 4, in the text in the Results section under subheading “Stage 3. Identify content and implementation options”, (pages 7-9), and subheading “Mapping the principle of the implementation intervention by form and function” (page 10). | _____________ |
|  | **WHO PROVIDED** |  |  |
| **5.** | For each category of intervention provider (e.g. psychologist, nursing assistant), describe their expertise, background and any specific training given. | Only planned delivery is reported in this paper. See Results section, subheading “Stage 3. Identify content and implementation options” (pages 7-9), and the published Study Protocol: https://doi.org/10.1186/s13012-021-01092-5. | _____________ |
|  | **HOW** |  |  |
| **6.** | Describe the modes of delivery (e.g. face-to-face or by some other mechanism, such as internet or telephone) of the intervention and whether it was provided individually or in a group. | Only planned delivery is reported in this paper. Procedures and modes of delivery are included in Table 3, Table 4 and in the Results section under sub-heading “Stage 3. Identify content and implementation options”, (pages 7-9) and subheading “Mapping the principle of the intervention by form and function” (page 10). | _____________ |
|  | **WHERE** |  |  |
| **7.** | Describe the type(s) of location(s) where the intervention occurred, including any necessary infrastructure or relevant features. | NA as this study does not report results of the larger trial. However, types of sites involved with the intervention are mentioned under sub-heading “Design and setting” on pages 5-6; and further information around planned implementation intervention delivery is included in Table 3, Table 4 and in the Results section subheading “Identify mode of delivery” (pages 8-9) and subheading “Mapping the principle of the implementation intervention by form and function” (page 10). | _____________ |
|  | **WHEN and HOW MUCH** |  |  |
| **8.** | Describe the number of times the intervention was delivered and over what period of time including the number of sessions, their schedule, and their duration, intensity or dose. | NA as this study does not report results of the larger trial. However, planned delivery is included in Table 3, Table 4 and in the results section under sub-heading “Stage 3. Identify content and implementation options”, (pages 7-9) and subheading “Mapping the principle of the intervention by form and function” (page 10). | _____________ |
|  | **TAILORING** |  |  |
| **9.** | If the intervention was planned to be personalised, titrated or adapted, then describe what, why, when, and how. | The implementation intervention is intended to be tailored, therefore standardised by function rather than form - see Table 1, Table 3, Table 4 and the Results section under sub-heading “Identify mode of delivery” (pages 8-9) and subheading “Mapping the principle of the implementation intervention by form and function” (page 10). | _____________ |
|  | **MODIFICATIONS** | NA as this study does not report results of the larger trial, however the rating and review of implementation strategies with key stakeholders resulted in confirmation of strategies following minor adjustments to their descriptions (see Table 1). |  |
| **10.^ǂ^** | If the intervention was modified during the course of the study, describe the changes (what, why, when, and how). |  | _____________ |
|  | **HOW WELL** |  |  |
| **11.** | Planned: If intervention adherence or fidelity was assessed, describe how and by whom, and if any strategies were used to maintain or improve fidelity, describe them. | See details around implementation intervention principles included under subheading “Mapping the principle of the implementation intervention by form and function” (page 10), Tables 1 & 4, and details in the published Study Protocol https://doi.org/10.1186/s13012-021-01092-5. | _____________ |
| **12.^ǂ^** | Actual: If intervention adherence or fidelity was assessed, describe the extent to which the intervention was delivered as planned. | NA – this paper does not report on results of the larger trial. | _____________ |

** **Authors** - use N/A if an item is not applicable for the intervention being described. **Reviewers** – use ‘?’ if information about the element is not reported/not sufficiently reported.

† If the information is not provided in the primary paper, give details of where this information is available. This may include locations such as a published protocol or other published papers (provide citation details) or a website (provide the URL).

ǂ If completing the TIDieR checklist for a protocol, these items are not relevant to the protocol and cannot be described until the study is complete.

* We strongly recommend using this checklist in conjunction with the TIDieR guide (see *BMJ* 2014;348:g1687) which contains an explanation and elaboration for each item.

* The focus of TIDieR is on reporting details of the intervention elements (and where relevant, comparison elements) of a study. Other elements and methodological features of studies are covered by other reporting statements and checklists and have not been duplicated as part of the TIDieR checklist. When a **randomised trial** is being reported, the TIDieR checklist should be used in conjunction with the CONSORT statement (see [www.consort-statement.org](http://www.consort-statement.org)) as an extension of **Item 5 of the CONSORT 2010 Statement.** When a **clinical trial** **protocol** is being reported, the TIDieR checklist should be used in conjunction with the SPIRIT statement as an extension of **Item 11 of the SPIRIT 2013 Statement** (see [www.spirit-statement.org](http://www.spirit-statement.org)). For alternate study designs, TIDieR can be used in conjunction with the appropriate checklist for that study design (see [www.equator-network.org](http://www.equator-network.org)).

**Article title:** Building staff capability, opportunity, and motivation to provide smoking cessation to people with cancer in Australian cancer treatment centres: development of an implementation intervention framework for the Care to Quit cluster randomised controlled trial.

**Journal name:** Health Services and Outcomes Research Methodology

**Author names:** Annika Ryan1,2 ORCID: **0000-0002-0487-7403;** Dr Alison Luk Young1 ORCID: 0000-0002-0810-4256; Jordan Tait1,2; Dr Kristen McCarter1,2,3,4 ORCID: [0000-0002-2638-6381](http://orcid.org/0000-0002-2638-6381); Melissa McEnallay1,2,3,4; Dr Fiona Day1,4,5; James McLennan6; Associate Professor Catherine Segan7,8; Gillian Blanchard5,14; Laura Healey5; Sandra Avery9,11; Dr Sarah White10; Professor Shalini Vinod11,12; Linda Bradford13; *Professor Christine L Paul1,2,3,4 ORCID: [**0000-0002-0504-5246**](https://orcid.org/0000-0002-0504-5246)

**Author affiliations:**

1. University of Newcastle, School of Medicine and Public Health, College of Health, Medicine and Wellbeing, Callaghan, New South Wales, Australia
2. Level 4 West, Hunter Medical Research Institute, John Hunter Hospital, New Lambton Heights, Newcastle, New South Wales, Australia
3. University of Newcastle, Priority Research Centre for Cancer Research, Innovation and Translation, Callaghan New South Wales 2308, Australia
4. Hunter Cancer Research Alliance, John Hunter Hospital, New Lambton Heights, Newcastle, New South Wales, Australia
5. Calvary Mater Newcastle, Corner Edith and Platt Streets, Waratah, New South Wales 2289, Australia
6. St Vincent’s Hospital Sydney, 390 Victoria Street, Darlinghurst, New South Wales 2010, Australia
7. Cancer Council Victoria, Melbourne, Victoria, Australia
8. Centre for Health Policy, Melbourne School of Population and Global Health, The University of Melbourne, Melbourne, Victoria, Australia
9. South Western Sydney Local Health District, Elizabeth Street, Liverpool New South Wales 2170, Australia
10. Department of Health Quitline, 615 St Kilda Rd, Melbourne Victoria, 3004
11. Cancer Therapy Centre, Liverpool Hospital, Liverpool New South Wales, Australia
12. South Western Sydney Clinical School and Ingham Institute for Applied Medical Research, Liverpool, New South Wales, Australia
13. The Alfred, 55 Commercial Rd, Melbourne, Victoria 3004, Australia
14. University of Newcastle, School of Nursing and Midwifery, Callaghan, New South Wales, Australia

**Email address of corresponding author**: [Chris.Paul@newcastle.edu.au](mailto:Chris.Paul@newcastle.edu.au)
